# Deep Learning Analysis of Mammography for Breast Cancer Risk Prediction in Asian Women

**DOI:** 10.3390/diagnostics13132247

**Published:** 2023-07-03

**Authors:** Hayoung Kim, Jihe Lim, Hyug-Gi Kim, Yunji Lim, Bo Kyoung Seo, Min Sun Bae

**Affiliations:** 1 Department of Radiology, College of Medicine, Inha University Hospital, Inhang-ro 27, Jung-gu, Incheon 22332, Republic of Korea; gloria21rad@gmail.com; 2 Department of Radiology, Hallym University Dongtan Sacred Heart Hospital, Hwaseong-si 18450, Gyeonggi-do, Republic of Korea; 3 Department of Radiology, Kyung Hee University Hospital, Seoul 02447, Republic of Korea; khyukgi@gmail.com; 4 Department of Radiology, Korea University Ansan Hospital, Korea University College of Medicine, Ansan-si 15355, Gyeonggi-do, Republic of Korea

**Keywords:** deep learning, mammography, risk prediction, breast cancer

## Abstract

The purpose of this study was to develop a mammography-based deep learning (DL) model for predicting the risk of breast cancer in Asian women. This retrospective study included 287 examinations in 153 women in the cancer group and 736 examinations in 447 women in the negative group, obtained from the databases of two tertiary hospitals between November 2012 and March 2022. All examinations were labeled as either dense breast or nondense breast, and then randomly assigned to either training, validation, or test sets. DL models, referred to as image-level and examination-level models, were developed. Both models were trained to predict whether or not the breast would develop breast cancer with two datasets: the whole dataset and the dense-only dataset. The performance of DL models was evaluated using the accuracy, precision, sensitivity, specificity, F1 score, and area under the receiver operating characteristic curve (AUC). On a test set, performance metrics for the four scenarios were obtained: image-level model with whole dataset, image-level model with dense-only dataset, examination-level model with whole dataset, and examination-level model with dense-only dataset with AUCs of 0.71, 0.75, 0.66, and 0.67, respectively. Our DL models using mammograms have the potential to predict breast cancer risk in Asian women.

## 1. Introduction

Breast cancer is the most commonly diagnosed cancer and the leading cause of cancer deaths in women worldwide [1]. Mammography is the primary imaging modality for breast cancer screening. Randomized controlled trials and incidence-based studies have reported the benefits of routine mammography screening in reducing breast cancer mortality [2,3].

However, a “one-size-fits-all” approach for screening may have the risk of underdiagnosis of breast cancer, especially in women with dense breasts. The sensitivity of mammography depends on breast density, which refers to the amount of fibroglandular tissue compared with that of fatty tissue in the breast [4,5,6,7]. According to the Breast Cancer Surveillance Consortium, the sensitivity of mammography decreased from 86–89% in women with fatty breasts to 62–68% in women with dense breasts [5,8]. More importantly, mammographic breast density is an independent risk factor for breast cancer [9,10]. Studies have shown an association between dense breast tissue and increased risk of breast cancer in both Western and Asian women [11,12,13]. Women with extremely dense breasts are 4–6 times more likely to develop breast cancer compared with those with fatty breasts [5,9,14].

In recent decades, several risk prediction models for breast cancer have been developed [15,16,17,18]. Initial studies were mainly based on clinical risk factors such as age at menarche, age at first childbirth, hormone replacement therapy use, and family history of breast cancer [18,19]. Because more recent studies have suggested that mammographic breast density is the strongest risk factor, breast density has been incorporated into the Gail model and the Tyrer–Cuzick model, which has resulted in improvement in model performance [20,21].

With the development of artificial intelligence (AI), studies have applied a deep learning algorithm to breast cancer risk assessment using information on mammograms [22,23,24,25]. A mammography-based deep learning risk model has shown superior accuracy in predicting breast cancer risk compared with traditional risk models across seven global institutions [22,23]. However, the existing risk prediction models were developed based on predominantly Western populations [17,18,19,20,21,22,24,25], and there are insufficient data on risk assessment models for Asian women. It is well known that the breast density and age-specific incidence of breast cancer differ between women in Asian and Western countries. High breast density is more frequent in Asian women compared with Western women [26,27]. In East Asian women, the peak age of breast cancer is in their 40s and 50s, while in Western women, it is in their 60s and 70s [28].

Given the differences in mammographic and clinical characteristics between Asian and Western women, developing a risk prediction model for Asian women is needed. The purpose of this study was to develop a deep learning model based on mammograms alone for predicting breast cancer risk in Asian women.

## 2. Materials and Methods

### 2.1. Data Collection

This retrospective study was approved by the Institutional Review Board of our institution (IRB No. 2020-02-033-003) and the requirement for written informed consent was waived. We collected consecutive digital mammograms between November 2012 and March 2022 at two tertiary hospitals. We also obtained data on patients with breast cancer from electronic medical records and pathology reports. The cancer group included mammograms before cancer diagnosis in patients with breast cancer diagnosed at each hospital. We excluded mammograms within 1 year before breast cancer diagnosis and women with a prior history of breast cancer in the same breast. However, women with a history of contralateral breast cancer were included. The negative group included all screening mammograms in women who had at least 5-year follow-up. Only mammograms with negative or benign results (Breast Imaging and Reporting Data System [BI-RADS] category 1 or 2) were included based on the mammography report in both groups. If each woman had multiple mammographic examinations during the study period, each examination was independently included as the index mammography. In addition, 17 examinations were excluded due to technical issues in image preprocessing for deep learning.

Finally, our study included 287 examinations in 153 women (hospital A, 189 examinations in 96 women; hospital B, 98 examinations in 57 women) in the cancer group and 736 examinations in 447 women (hospital A, 252 examinations in 161 women; hospital B, 484 examinations in 286 women) in the negative group.

### 2.2. Mammographic Examinations and Data Categorization

Full-field digital mammography was acquired using two different machines (Hologic and GE HealthCare) at two hospitals. Standard mammography included craniocaudal and mediolateral oblique views of each breast. The final assessment category and breast density based on the BI-RADS system were determined based on the mammography report [29]. We classified all mammographic examinations into two categories (dense and nondense) based on the BI-RADS density grading system. The BI-RADS grades 1 or A (almost entirely fatty) and 2 or B (scattered areas of fibroglandular tissue) were considered nondense, and the BI-RADS grades 3 or C (heterogeneously dense) and 4 or D (extremely dense) were considered dense.

**Figure 1:**
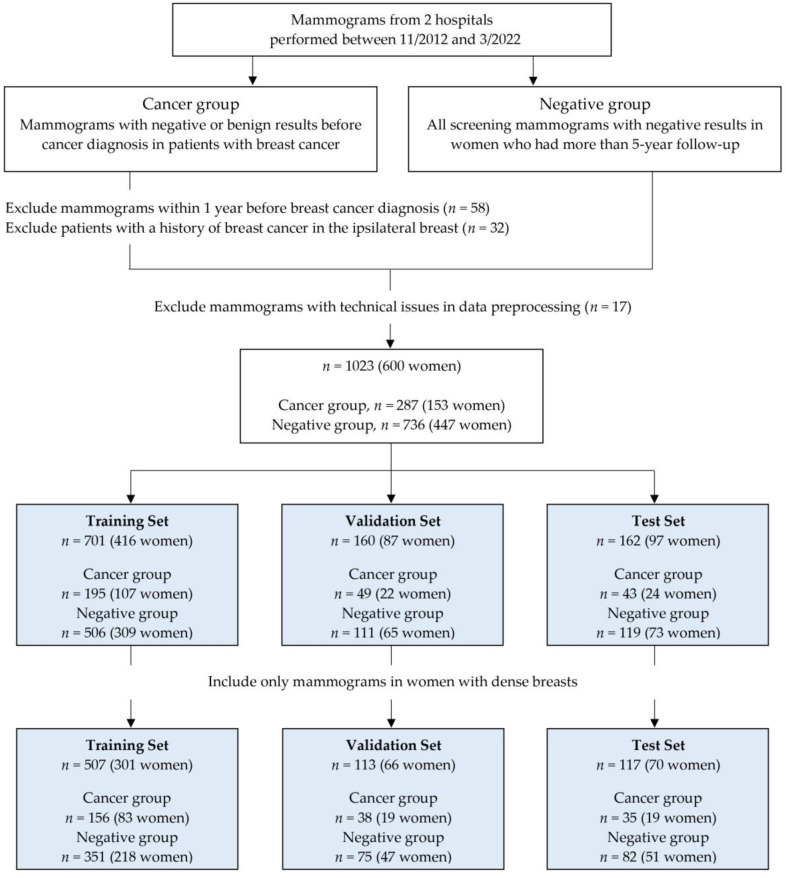
Flowchart shows data inclusion and exclusion criteria for the training, validation, and test sets.

All mammograms were saved as digital imaging and communications in medicine (DICOM) images and uploaded to the cloud system for data processing. In the cancer group, only images of the breast where cancer occurred were uploaded for each examination. In the negative group, all four full-field digital mammograms were uploaded. All images were labeled as “cancer” or “negative” and “dense” or “nondense.” After image preprocessing, all mammograms were randomly assigned to either training, validation, or test sets. We split the dataset by women, so each woman only contributed mammograms to one set. A flowchart illustrating the construction and distribution of whole datasets is shown in Figure 1.

### 2.3. Model Development

In this study, we prepared the two separate datasets to investigate whether prior mammograms could predict a risk of breast cancer: the whole dataset, which included all mammograms in women with dense and nondense breasts; and the dense-only dataset, which included mammograms only in women with dense breasts. The deep convolutional neural networks were, respectively, trained using the whole and dense-only datasets. In addition, deep learning models were trained based on both the examinations (comprising two images per breast) and the images.

We used commercially available software (Neuro-X v3.0.1, Neurocle Inc., Seoul, Republic of Korea) to train the two deep learning models—that is, image-level and examination-level models. Data augmentation options, which included the image rotation at 90° and the image flipping process for horizontal and vertical directions, were randomly applied as follows: hue at –0.1 to 0.1, brightness at –0.12 to 0.12, contrast at 0.6 to 1.4, and flipping process for horizontal and vertical directions.

All training processes were performed on a single workstation computer with the Windows operating system (Windows 10, Microsoft, 2015) and using the NVIDIA Quadro RTX 8000 with 48 GB of memory (Nvidia Corporation, Santa Clara, CA, USA).

### 2.4. Statistical Analysis

The deep learning models separately evaluated each image. However, individual image results were combined as the outcome of a mammographic examination. The trained model was analyzed by mammographic images and examinations to evaluate the performance of the deep learning model. For analysis based on mammographic images, the result was considered as cancer if an image predicted a developing breast cancer. For analysis based on mammographic examinations, the result was considered as cancer if at least one image predicted a developing breast cancer. Otherwise, the result was considered as negative. Consequently, four deep learning models were obtained: an image-level model with the whole dataset, an image-level model with the dense-only dataset, an examination-level model with the whole dataset, and an examination-level model with the dense-only dataset. For each scenario, the model performance was evaluated with the accuracy, precision, sensitivity, specificity, F1 score, and area under the receiver operating characteristic (ROC) curve (AUC). A two-sided 95% confidence interval (CI) was obtained for the sensitivity, specificity, and AUC.

## 3. Results

### 3.1. Characteristics of the Study Sample

A total of 1023 mammographic examinations in 600 women (mean age, 52 years; range, 31–84 years) were included, and we randomly assigned to training (701 examinations in 416 women), validation (160 examinations in 87 women), and test (162 examinations in 97 women) sets. The dense-only dataset consisted of 737 examinations in 437 women with dense breasts. This dataset was divided into training (507 examinations in 301 women), validation (113 examinations in 66 women), and test (117 examinations in 70 women) sets (Figure 1).

Table 1 shows the clinical characteristics of the three sets. The proportion of dense breasts in the whole dataset was 72.0% (737 of 1023), consisting of 79.8% (229 of 287) in the cancer group and 69.0% (508 of 736) in the negative group. Of the 861 mammographic examinations used for training and validation, 244 (28.3%) were classified as the cancer group. Of the 162 mammographic examinations used for testing, 43 (26.5%) were classified as the cancer group. The vast majority of cancers diagnosed were invasive carcinoma (88.5% (254 of 287)).

### 3.2. Performance of Risk Prediction Models

We evaluated the performance of mammography-based deep learning models for predicting breast cancer risk. The performance measures of the risk prediction models on test sets are summarized in Table 2.

**Figure 2:**
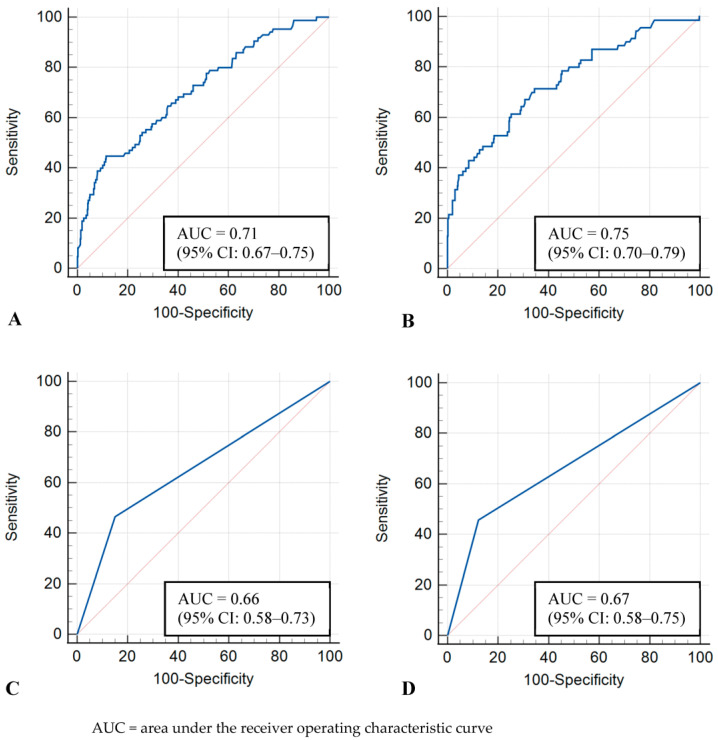
Receiver operating characteristic curves for test sets. (**A**) Image-level model with whole dataset, (**B**) Image-level model with dense-only dataset, (**C**) Examination-level model with whole dataset, (**D**) Examination-level model with dense-only dataset.

The image-level model showed AUCs of 0.71 (95% CI: 0.67–0.75) and 0.75 (95% CI: 0.70–0.79) in the whole and dense-only datasets, respectively. The examination-level model showed AUCs of 0.66 (95% CI: 0.58–0.73) and 0.67 (95% CI: 0.58–0.75) in the whole and dense-only datasets, respectively. No statistically significant difference was observed between the image-level and examination-level models (whole, *p* = 0.17; dense-only, *p* = 0.16). The ROC curves of the four models are shown in Figure 2.

The image-level and examination-level models showed similar sensitivity and specificity in the whole dataset (sensitivity, 44.7% [38 of 85; 95% CI: 33.9–55.9] vs. 46.5% [20 of 43; 95% CI: 31.2–62.3]; specificity, 81.6% [386 of 473; 95% CI: 77.8–85.0] vs. 84.9% [101 of 119; 95% CI: 77.2–90.8]) and the dense-only dataset (sensitivity, 41.4% [29 of 70; 95% CI: 29.8–53.8] vs. 45.7% [16 of 35; 95% CI: 28.8–63.4]; specificity, 91.5% [300 of 328; 95% CI: 87.9–94.3] vs. 87.8% [72 of 82; 95% CI: 78.7–94.0]). In terms of the precision and F1 score, the examination-level model was superior to the image-level model in the whole dataset (precision, 52.6 [20 of 38] vs. 30.4 [38 of 125]; F1 score, 0.494 vs. 0.362) and the dense-only dataset (precision, 61.5 [16 of 26] vs. 50.9 [29 of 57]; F1 score, 0.525 vs. 0.457).

## 4. Discussion

In this study, we developed a deep learning model using full-field digital mammograms to predict breast cancer risk in Asian women with a high proportion of dense breasts. The image-level and examination-level deep learning models were created and trained with whole and dense-only datasets, respectively. On a test set, the mammography-based risk models showed a reasonable performance in predicting the risk of breast cancer (AUC, 0.66–0.75).

The AUC values of our model were comparable with those of existing image-based risk models [22,23,24]. Yala et al. [22] developed a deep learning model that used mammograms in addition to traditional risk factors (a hybrid deep learning model) to assess breast cancer risk. Although the hybrid model was the best model (AUC, 0.70), a deep learning model based on mammograms alone outperformed the Tyrer–Cuzick model (AUC, 0.68 vs. 0.62). This finding suggests that the mammography-based risk model can provide breast cancer risk assessment when traditional risk factor information is unavailable. Compared with the mammography-based risk model (AUC, 0.65–0.74) developed by Eriksson et al. [24], our risk assessment models achieved similar performance (AUC, 0.66–0.75).

The majority of image-based risk models were developed on predominantly white populations, and thus have limitations in predicting risk for Asian women [22,24,25]. In contrast, our risk models were targeted for only Asian women (Korean women). There is a distinct age distribution of breast cancer among Asian women compared with white women. The incidence of breast cancer among Asian women peaks at age 45–49 years, whereas breast cancer incidence peaks among non-Hispanic white women at age 75–79 years [28]. It has also been shown that mammographic parenchymal patterns differ between Asian and Western women [26,30]. In a study of mammography data on more than one million women [30], Asian women had the highest proportion of dense breast tissue compared with other racial groups. High breast density reduces the sensitivity of screening mammography and can increase the incidence of interval breast cancer because overlapping fibroglandular dense tissue can mask a breast lesion [9]. In addition, breast density itself is a strong risk factor for developing breast cancer [10,11,12].

Simulation modeling studies have shown that screening strategies should be personalized based on a woman’s age, breast density, and other risk factors [6,31]. Individual women have different needs for breast cancer screening. All women can be assessed for breast cancer risk based on AI-based or traditional risk models, and then be stratified into average-, moderate-, and high-risk groups [32]. Personalized risk-based screening may be more important for average-risk women with dense breasts to identify the potential candidates for supplemental screening and more frequent screening. Lehman et al. [25] compared the performance of a deep learning image-based risk model with traditional risk models in the screening setting, and found that the deep learning score derived from the woman’s prior mammogram outperformed traditional risk models in identifying the subgroup of women with higher cancer burden.

In this study, both image-level and examination-level deep learning models performed better on the dense-only dataset compared with the whole dataset in all performance measures except sensitivity. Our study populations had a high percentage of dense breast tissue. In the whole dataset, the proportions of dense breasts were 72.3% (507 of 701) and 70.6% (113 of 160) in the training and validation sets, respectively.

Our study had several limitations. First, this was a retrospective study with a relatively small sample size. Because we used mammography data obtained from only two institutions, the generalizability of our results may be limited. If a deep learning model is trained with a large dataset collected from multiple institutions, the model performance could be further improved. Second, negative or benign results from the mammography report might include missed or subtle cancers. However, we did not include mammograms within 1 year before breast cancer diagnosis in the cancer group. Lastly, our deep learning models were developed based on the patient’s mammographic images alone. The predictive accuracy of deep learning risk models can be further improved by incorporating the genetic test result and clinical risk factors such as age, menopausal status, family history of breast cancer, prior benign biopsy, or use of hormone therapy. However, the mammography-based deep learning model has advantages because it does not require knowledge of those risk factors required by traditional risk models.

In conclusion, we developed a deep learning risk model based on the mammography data from Asian women. Our deep learning model demonstrated comparable predictive accuracy in breast cancer risk assessment compared with existing risk models. Further studies are required to validate our model across institutions in larger datasets. The mammography-based risk model has the potential to support effective risk-based screening in clinical practice.

## Figures and Tables

**Table 1:** Characteristics of the Training, Validation, and Test Sets.

Characteristics	Training Examinations		Validation Examinations		Test Examinations	
	(*n* = 701)		(*n* = 160)		(*n* = 162)	
	Negative	Cancer	Negative	Cancer	Negative	Cancer
	(*n* = 506)	(*n* = 195)	(*n* = 111)	(*n* = 49)	(*n* = 119)	(*n* = 43)
Age (years)						
<40	39 (7.7)	15 (7.7)	14 (12.6)	3 (6.1)	12 (10.1)	2 (4.7)
40–50	204 (40.3)	75 (38.5)	37 (33.3)	10 (20.4)	36 (30.3)	27 (62.8)
51–60	138 (27.3)	60 (30.8)	32 (28.8)	26 (53.1)	41 (34.5)	9 (20.9)
>60	125 (24.7)	45 (23.1)	28 (25.2)	10 (20.4)	30 (25.2)	5 (11.6)
Mean ± SD	51 ± 10	53 ± 11	51 ± 10	54 ± 9	53 ± 10	48 ± 7
Breast density						
Almost entirely fatty	48 (9.5)	0 (0)	12 (10.8)	1 (2.0)	9 (7.6)	1 (2.3)
Scattered areas of FGT	107 (21.1)	39 (20.0)	24 (21.6)	10 (20.4)	28 (23.5)	7 (16.3)
Heterogeneously dense	276 (54.5)	114 (58.5)	58 (52.3)	29 (59.2)	60 (50.4)	22 (51.2)
Extremely dense	75 (14.8)	42 (21.5)	17 (15.3)	9 (18.4)	22 (18.5)	13 (30.2)
Nondense vs. dense						
Nondense breasts	155 (30.6)	39 (20.0)	36 (32.4)	11 (22.4)	37 (31.1)	8 (18.6)
Dense breasts	351 (69.4)	156 (80.0)	75 (67.6)	38 (77.6)	82 (68.9)	35 (81.4)
Histologic type						
Invasive carcinoma	-	172 (88.2)	-	46 (93.9)	-	36 (83.7)
DCIS	-	23 (11.8)	-	3 (6.1)	-	7 (16.3)

Numbers are raw data and percentages are in parentheses. SD = standard deviation, FGT = fibroglandular tissue, DCIS = ductal carcinoma in situ.

**Table 2:** Performance of Deep Learning Risk Prediction Models on Test Sets.

Metrics	Image-Level Model		Examination-Level Model	
	Whole	Dense-Only	Whole	Dense-Only
Accuracy (%)	76.0 (424 of 558)	82.7 (329 of 398)	74.7 (121 of 162)	75.2 (88 of 117)
Precision (%)	30.4 (38 of 125)	50.9 (29 of 57)	52.6 (20 of 38)	61.5 (16 of 26)
Sensitivity (%)	44.7 (38 of 85)	41.4 (29 of 70)	46.5 (20 of 43)	45.7 (16 of 35)
Specificity (%)	81.6 (386 of 473)	91.5 (300 of 328)	84.9 (101 of 119)	87.8 (72 of 82)
F1 score	0.362	0.457	0.494	0.525

## Data Availability

Data will be provided upon request.
